# Efficacy of single dose of gentamicin in combination with metronidazole versus multiple doses for prevention of post-caesarean infection: study protocol for a randomized controlled trial

**DOI:** 10.1186/1745-6215-13-89

**Published:** 2012-06-21

**Authors:** Fadhili M Lyimo, Anthony N Massinde, Benson R Kidenya, Evelyne Konje, Stephen E Mshana

**Affiliations:** 1 Department of Obstetrics and Gynaecology, Catholic University of Health Sciences and Allied Science and Bugando Medical Centre, Box 1464, Mwanza, Tanzania; 2 Department of Biochemistry, Catholic University of Health Sciences and Allied Sciences, Mwanza, Tanzania; 3 Department of Epidemiology and Biostatistics, Catholic University of Health Sciences and Allied Sciences, Mwanza, Tanzania; 4 Department of Microbiology/Immunology, Catholic University of Health Sciences and Allied Sciences, Mwanza, Tanzania

**Keywords:** Post-caesarean infection, Metronidazole, Gentamicin, Mwanza, Tanzania

## Abstract

**Background:**

Caesarean section is a commonly performed operation worldwide. It has been found to increase rates of maternal infectious morbidities more than five times when compared to vaginal delivery. Provision of intravenous prophylactic antibiotics 30 to 60 minutes prior to caesarean section has been found to reduce post-caesarean infection tremendously. Many centers recommend provision of a single dose of antibiotics, as repeated doses offer no benefit over a single dose.

At Bugando Medical Centre post caesarean infection is among the top five causes of admission at the post-natal ward. Unfortunately, there is no consistent protocol for the administration of antibiotic prophylaxis to patients who are designated for caesarean section. Common practice and generally the clinician’s preference are to provide repeated dosages of antibiotic prophylaxis after caesarean section to most of the patients. This study aims to determine the comparative efficacy of a single dose of gentamicin in combination with metronidazole versus multiple doses for prevention of post caesarean infection.

**Methods/Design:**

The study is an interventional, open-label, two-armed, randomized, single-center study conducted at Bugando Medical Centre Mwanza, Tanzania. It is an ongoing trial for the period of seven months; 490 eligible candidates will be enrolled in the study. Study subjects will be randomly allocated into two study arms; “A” and “B”. Candidates in “A” will receive a single dose of gentamicin in combination with metronidazole 30 to 60 minutes prior to the operation and candidates in “B” will receive the same drugs prior to the operation and continue with gentamicin and metronidazole for 24 hours. The two groups will be followed up for a period of one month and assessed for signs and symptoms of surgical site infection.

Data will be extracted from a case record form and entered into Epi data3.1 software before being transferred to SPSS version 17.0 for analysis. The absolute difference in proportion of women who develop surgical site infection in the two study arms will be the effectiveness of one regime over the other.

**Trial registration:**

Current Controlled TrialsISRCTN44462542.

## Background

Postpartum infection remains among the top five causes of pregnancy-related maternal mortality and morbidity worldwide [1-3]. Women who undergo caesarean section have a 5- to 20-fold greater risk of postpartum infection than women having a vaginal delivery [4-7]. The incidence of post-cesarean infection varies widely worldwide from 2.5% to 20.5% [8-12].

Post-caesarean infections are polymicrobial, involving aerobes, anaerobes and ureaplasma. The main source of postpartum infection after caesarean section is the lower genital tract, particularly if the membranes are ruptured, but this still occurs with intact membranes following preterm birth. The most common isolated pathogens are anaerobes and gram-negative aerobes. Gram-negative aerobes include *Escherichia coli, Klebsiella* spp, *Enterobacter* spp. and *Proteus* spp*.* The anaerobes include *Bacteroides* spp*., Clostridium* spp.*,* and *Fusobacterium* spp*.*[8-10]*.* However, exogenous bacterial contamination by skin flora (such as *Staphylococcus aureus*) as a result of a break in sterile technique, may occur, especially following a difficult or complicated surgery [11]. Mawalla *et al.*, in a prospective cross-sectional study, reported that most common isolates in surgical site infection at Bugando Medical Centre are gram-negative bacteria. *Staphylococcus aureus* was found in only 28.6% of study patients; and 18.8% were MRSA (Methicillin resistant *Staphylococcus aureus*) [12].

Selection of antibiotics for prophylaxis follows the principle that the selected antibiotic regimen should have activity against the microbial agent commonly involved in surgical site contamination and actual infection. A combination of clindamycin and aminoglycosides has been recommended as the treatment of choice in post-caesarean infection, since it covers most of the pathogenic bacteria commonly involved. Alternatively, a combination of metronidazole with aminoglycosides has been found to be effective in some studies [8,10,13,14]. Apart from being a drug of choice in treating gram-negative bacteria, methicillin sensitive *Staphylococcus aureus* responds to gentamicin [14-16].

A number of well-designed studies have documented the efficacy of prophylactic antibiotics in reducing the rates of post-partum infection among patients who have undergone caesarian section [17-21]. Administration of antibiotic prophylaxis within an hour prior to skin incision is more effective in reducing post-caesarean infectious morbidity when compared to administration of the same drugs after cord clamping, and has no effect on neonatal infection [22-26]. Owens *et al.*, in a systematic review, reported that provision of antibiotic prophylaxis for caesarean section before skin incision compared with after umbilical cord clamping is associated with a 40% decrease in postpartum endometritis and a 30% decrease in wound infection [27].

Provision of a single dose of antibiotics preoperatively has been found to be as effective as multiple doses, in prevention of postpartum infection [28,29].

At Bugando Medical Centre (BMC), the rate of caesarian is estimated to be 20% [30]. All patients who deliver by caesarian section receive antibiotic prophylaxis. There is no consistent protocol for administration of antibiotic prophylaxis for patients designated for caesarean section The common practice is to provide repeated doses of prophylactic antibiotics for at least 24 hours after caesarian section and often on individual clinicians’ practice and preference. Gentamicin and metronidazole are among the antibiotics which are used as prophylaxis for infection during caesarean delivery.

This study aims to compare the efficacy of an intravenous single dose of gentamicin (3 mg/Kg) plus metronidazole 500 mg given 30 to 60 minutes before incision and multiple doses of gentamicin (3 mg/Kg) plus metronidazole (500 mg) 30 to 60 minutes before incision and every 8 hours for 24 hours postoperatively.

### Justification for this study

Caesarean section remains an important risk factor for post-partum infection in many health facilities. Surgical site infection post-caesarean section is among the top five causes of admission in the postnatal ward at Bugando Medical Centre. There is no consistent protocol for provision of prophylactic antibiotics at our obstetric unit. Therefore, the findings from this study will help to improve the quality of care by using standard protocol for antibiotic prophylaxis among patients undergoing caesarean section.

### Study hypothesis

In this study, the hypothesis is: “there is no clinically significant difference between a single dose of gentamicin in combination with metronidazole and multiple doses over 24 hours for prevention of post caesarean infection".

## Methods/Design

### Study design and site

This is an interventional, open label, two-armed, randomized, single center study to be conducted at BMC, a zonal consultant and teaching hospital situated in Mwanza City, Tanzania. In this hospital, 7,149 pregnant women are admitted yearly. There are 6,868 deliveries per annum, 1,398 (20.3%) pregnant women deliver by caesarean section [30].

### Targeted population

The targeted study population is all pregnant women who are admitted at the BMC labor ward and planned for emergency caesarean section.

### Sample size estimation

The incidence of surgical site infection ranges between 2.5% and 20%. Zelenitsky *et al.*, in his prospective randomized study, found that deep surgical site infection rates were 8.1% and 6.9% in the single high dose and multiple standard dose groups, respectively [31].

For the purpose of this study, which involves candidates who undergo emergency or non-elective caesarean section, we use Ps = 8.1%, which is the proportion of participants in the single dose antibiotic prophylaxis group expected to develop surgical site infection post caesarian section, and Pn = 6.9%, which is proportion of participants in the multiple dose antibiotic prophylaxis expected to develop surgical site infection. If we assume the hypothesized difference, D to be 5%, the sample size needed to reject this hypothesis at alpha = 0.05 and beta = 0.20 is [32]:

(1) $${\left({\text{Z}}_{0.95}\;\text{+}\;{\text{Z}}_{0.80}\right)}^{2}\;\left[\text{Ps}\left(1-\text{Ps}\right)\text{Pn}\;+\left(1-\text{Pn}\right)\right]/{\left(\text{Ps-Pn-D}\right)}^{2}$$

Substituting to the formula, the total required sample size: 444 (that is, the required sample size in each group will be 222).

When considering 10% of participants drop out or are lost to follow-up, the required sample size will be 490; that is, each group will contain 245 participants.

### Eligibility/Enrollment

After the decision for caesarean section is made, the pregnant woman will be assessed to see if she is eligible for this study. The assessment for eligibility will be based on inclusion and exclusion criteria given below.

#### Inclusion criteria

All pregnant women who planned for caesarean section and have consented for the study are eligible for inclusion in the study.

#### Exclusion criteria

All pregnant women with fever (temperature of 38°C and above), prolonged obstructed labor and premature rupture of membranes (rupture of membrane more than 12 hours will be excluded. Pregnant women presenting with features of chorioamnionitis (that is, foul smelling lochia, uterine tenderness associated with fever), allergic to the antibiotics used in the study or those who have used antibiotics in the 24 hours preceding the operation will also be excluded.

### Randomization

Intervention will start after allocating eligible candidates into two study arms: A and B.

Study arm A will be those who will receive a single intravenous single dose of gentamicin (3 mg/Kg) plus metronidazole 500 mg 30 to 60 minutes before operation. Study arm B will be those who will receive multiple doses of gentamicin (3 mg/Kg) plus metronidazole (500 mg) 30 to 60 minutes before operation and metronidazole 500 mg every 8 hours for 24 hours.

Simple randomization will be used to allocate study participants. About 490 opaque envelopes of the same size and color will be prepared for this study. A total of 245 envelopes will contain papers marked “study arm A” and the remaining envelopes will contain papers marked “study arm B”. Then all envelopes will be mixed thoroughly in a box before selection of an envelope is done.

Each study participant will select one sealed envelope and give it to the research assistant to open. Then, she will be administered antibiotic prophylaxis according to the study allocation group.

### Primary outcome measures

Surgical site infection will be our primary outcome - the assessment for any evidence of surgical site infection will be done 72 hours after caesarian section, as well as on follow-up days (Day 7 and Day 30 post-caesarean section). Two clinicians in the ward who are not aware of the study group allocation will perform diagnosis of surgical site infection. The presence of fever (febrile morbidity), signs and symptoms of abdominal wound infection or endometritis will indicate surgical site infection. Febrile morbidity will be defined by temperature above 38°C at least 4 hours apart on two or more occasions, excluding the first 24 hours after delivery [33]. Abdominal wound infection will be defined by partial or total dehiscence or presence of purulent or serous discharge from the wound with indurations, warmth and tenderness.

Endometritis will be defined by the presence of fever (38°C or above) in association with one or more of the following: uterine tenderness or foul smelling lochia [33]. In both groups, the bladder catheter will be removed after 24 hours. Wound care will follow the standard scheme in both groups, the occlusive dressing applied in the theatre and removed after 48 hours. The patient will be discharged on Day 3 if there is no sign of infection or complication and asked to return on Day 7 in order to have her stitches removed. Then, the patient will return on Day 30 post-caesarean section for reassessment. On Day 7 and Day 30, axillary temperature will be measured, and the abdomen and wound will be examined for signs of infection and sutures will be removed on the first follow-up visit.

### Secondary outcome measure

There are no secondary outcome measures.

### Data collection

All data will be extracted from patients’ files and filled in a case record form designed for this study. Data to be collected will include the woman’s socio-demographic characteristics, obstetric history (past and present), and labor history if any (stage of labor, state of amniotic membrane-whether intact or ruptured) and others. Information on the HIV status of all women will also be collected. Patients’ information will be added during follow up.

### Data management

Data will be entered in Epi data 3.1 software (Epidata Association, Odense, Denmark) and later transferred into SPSS version 17.0 (University of Chicago, Chicago, USA) for analysis. Cleaning will be done using the same package.

### Data analysis

Analysis will involve baseline and hypothesis testing. Baseline analysis will involve comparing of the baseline characteristics between the two study arms. Hypothesis testing will be done to determine if there is a significant difference in cumulative incidence of post-caesarian infection between women under single dose antibiotic regimen and those under the multiple-doses antibiotic regimen. The analysis will be done as per protocol. The absolute difference in the proportion of women who will develop surgical site infection in the two study arms will be the effectiveness of one regimen over the other. Results will be presented in cumulative incidence and as a relative ratio together with 95% confidence intervals and *P*-value. Sub-group analysis will be performed by logistic regression to determine the association between surgical site infection and other covariates, such as HIV status, duration of operation, stage of labor and so on. Covariates with *P*-value less than 0.05 will be considered significant. Efforts will be made to trace by calling those who will be lost to follow so as to determine their well-being, this information will be incorporated into the data collection form (Additional file 1).

### Quality control

The principal investigator will review data daily from case record forms (CRF) for completeness and consistence of the responses.

Two data clerks, ensuring that entered data quality will not rely on a single person, will conduct data entry. Where discrepancies are found, the two data entry clerks will be called together with the CRF to rectify the difference.

Participation refusal or the decline from study of a significant number of eligible women (more than 30%) may create uncertainty in the internal validity of the study.

Efforts will be made to explain to all eligible women the nature of the study and its importance for improving the care of women delivering by caesarean section at Bugando. We anticipate an attrition rate of 10% or less. Study participants will be reminded to attend their follow-up days through contact phone numbers provided by them.

### Data dissemination and utilization

Apart from being part of a dissertation proposal for FL, effort will be made to publish the final results. Importantly, the findings will be used to create a protocol for use of antibiotics as operative prophylaxis.

### Ethical approval

This trial does not involve new drugs, only timing of administration of drugs is being tested; however, Good Clinical Practice (GCP) and the Declaration of Helsinki are observed. Clearance was sought from the Bugando Research Ethics Committee on 22 August 2011, ref: BREC/001/39/2011.

An informed consent will be requested from participants after explaining the study aims. For literate women, the consent information will be provided followed by providing a copy of the consent form that each participant will be required to sign to signify her consent.

For non-literate women, the consent information sheet will be read in full and participants required to place a thumb print on the consent form to signify their acceptance to participate in the study. A nurse or doctor who is not part of the study will be around to witness and verifiy the counseling process for illiterate women.

Participation is voluntary and those declining to participate will still be entitled to the standard care provided to all women in the labor ward.

Regarding testing for HIV, the current policy is that every woman admitted at Bugando Medical Centre is requested to be tested for HIV. A trained counselor is available in the labor room to counsel all women coming for delivery. Testing is done according to the Tanzania Ministry of Health recommendation. The filled data collection form will be destroyed after data entry and cleaning.

## Trial status

The recruitment of patients started 1 October 2011, and it is expected that by 15 May 2012 the required sample size will be reached.

## Abbreviations

BMC, Bugando medical centre; CRF, Case record forms; GCP, Good clinical practice; MRSA, Methicillin resistant Staphylococcus aureus.

## Competing interests

The authors declare that they have no competing interests. Funding has been received from the Ministry of Health, United Republic of Tanzania as an unrestricted educational grant for a postgraduate thesis.

## Authors’ contributions

FL, AM, SEM, BK and EK have made considerable contributions to concept and design of this trial. They were all involved in drafting the study protocol, revising it critically for imperative intellectual content, and have given approval of the final version to be used in this trial. All authors read and approved the final manuscript.

## Supplementary Material

Additional file 1 Algorithm for Randomization. Click here for file
