# Supplementary figures and images for: Efficacy of single dose of gentamicin in combination with metronidazole versus multiple doses for prevention of post-caesarean infection: study protocol for a randomized controlled trial
Source: Trials. 2012 Jun 21;13:89. doi: 10.1186/1745-6215-13-89 (PMC3475059; doi:10.1186/1745-6215-13-89)

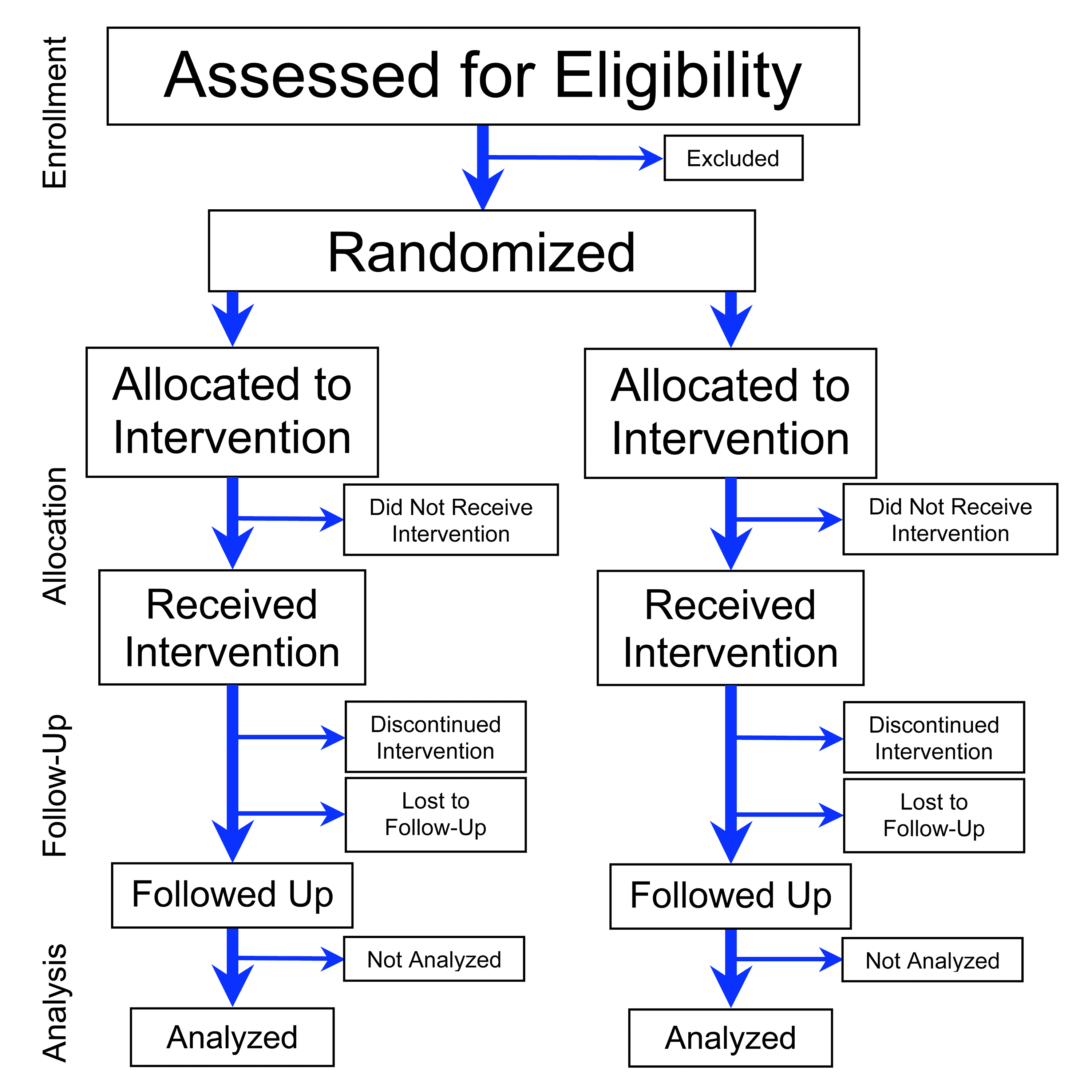

Supplement: Additional file 1 — Algorithm for Randomization. [file 1745-6215-13-89-S1.doc]
